# Torso-like mediates extracellular accumulation of Furin-cleaved Trunk to pattern the *Drosophila* embryo termini

**DOI:** 10.1038/ncomms9759

**Published:** 2015-10-28

**Authors:** Travis K. Johnson, Michelle A. Henstridge, Anabel Herr, Karyn A. Moore, James C. Whisstock, Coral G. Warr

**Affiliations:** 1 School of Biological Sciences, Monash University, Clayton, Victoria 3800, Australia; 2 Australian Research Council Centre of Excellence in Advanced Molecular Imaging, Monash University, Clayton, Victoria 3800, Australia; 3 Department of Biochemistry and Molecular Biology, Monash University, Clayton, Victoria 3800, Australia

## Abstract

Patterning of the *Drosophila* embryonic termini is achieved by localized activation of the Torso receptor by the growth factor Trunk. Governing this event is the perforin-like protein Torso-like, which is localized to the extracellular space at the embryo poles and has long been proposed to control localized proteolytic activation of Trunk. However, a protease involved in terminal patterning remains to be identified, and the role of Torso-like remains unknown. Here we find that Trunk is cleaved intracellularly by Furin proteases. We further show that Trunk is secreted, and that levels of extracellular Trunk are greatly reduced in *torso-like* null mutants. On the basis of these and previous findings, we suggest that Torso-like functions to mediate secretion of Trunk, thus providing the mechanism for spatially restricted activation of Torso. Our data represent an alternative mechanism for the spatial control of receptor signalling, and define a different role for perforin-like proteins in eukaryotes.

## Abstract (web summary)

Activation of the growth factor Trunk patterns the *Drosophila* embryonic termini but how this is regulated is unclear. Here, Johnson *et al.* report that Trunk is cleaved intracellularly by Furin proteases, and its extracellular accumulation is then mediated by the perforin-like protein Torso-like.

## Graphical abstract

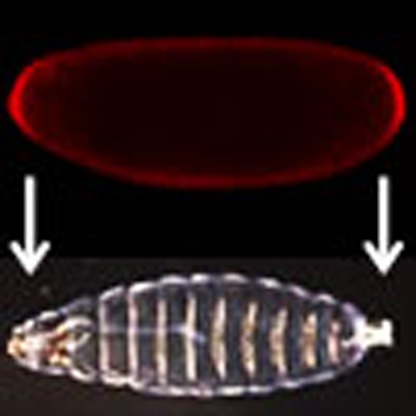

Controlled activation of cell receptors by extracellular growth factors governs embryonic patterning and development in eukaryotes. An archetypal example of the spatial control of receptor activation by a growth factor is seen in patterning of the *Drosophila* embryo termini. Here activation of the receptor tyrosine kinase Torso (Tor) occurs only at the termini, despite both Tor and its extracellular ligand Trunk (Trk) being ubiquitously expressed^1^,^2^,^3^,^4^. For many years it has been proposed that the cysteine-knot growth factor Trk is secreted and present ubiquitously in the perivitelline space (PVS) surrounding the embryo, and that its activation is controlled via localized proteolysis in the PVS in a manner analogous to the proteolytic cascade that activates the cysteine-knot growth factor Spätzle (Spz) during dorso-ventral patterning^2^,^3^,^5^. Genetic studies suggest that active Trk is generated in the polar PVS at rate-limiting quantities where it is efficiently captured by the Tor ectodomain^6^, however Trk localization has yet to be reported.

Currently the only known regionalized factor in terminal patterning is Torso-like (Tsl), which is associated with the vitelline membrane (VM) and embryonic plasma membrane in the polar PVS^7^,^8^,^9^,^10^,^11^,^12^. However, Tsl does not resemble any known protease, and rather belongs to the perforin-like superfamily of pore-forming proteins^13^. Tsl has therefore been proposed to control Trk cleavage indirectly, through controlling the activity of an unidentified protease^2^,^5^.

We recently confirmed that Trk is proteolytically cleaved and that this processing is essential for terminal patterning^14^. In contrast to the proposed model, however, we were unable to detect Trk cleavage products that were dependent on Tsl activity^14^. In spite of this, it remained possible that a localized effect of Tsl on Trk cleavage was too subtle for us to detect. Here to determine how Tor is activated, and the role of Tsl, we aimed to identify the protease(s) that cleave Trk and to further understand how and where these events occur. We reveal that, rather than being locally cleaved in the PVS, Trk is instead cleaved intracellularly before its secretion. We further demonstrate that Furin proteases are required for Trk cleavage, and show that the Furin 1 and Furin 2 proteases act redundantly in terminal patterning. Finally, we provide evidence that Trk is, as long believed, secreted into the PVS, and that this extracellular accumulation of Trk is greatly reduced in *tsl* mutants. On the basis of the present and other previous studies, we suggest that the function of Tsl is to mediate secretion of Trk, thus providing the mechanism for spatially restricted activation of Tor.

## Results

### Trk cleavage occurs intracellularly

**Figure 1:**
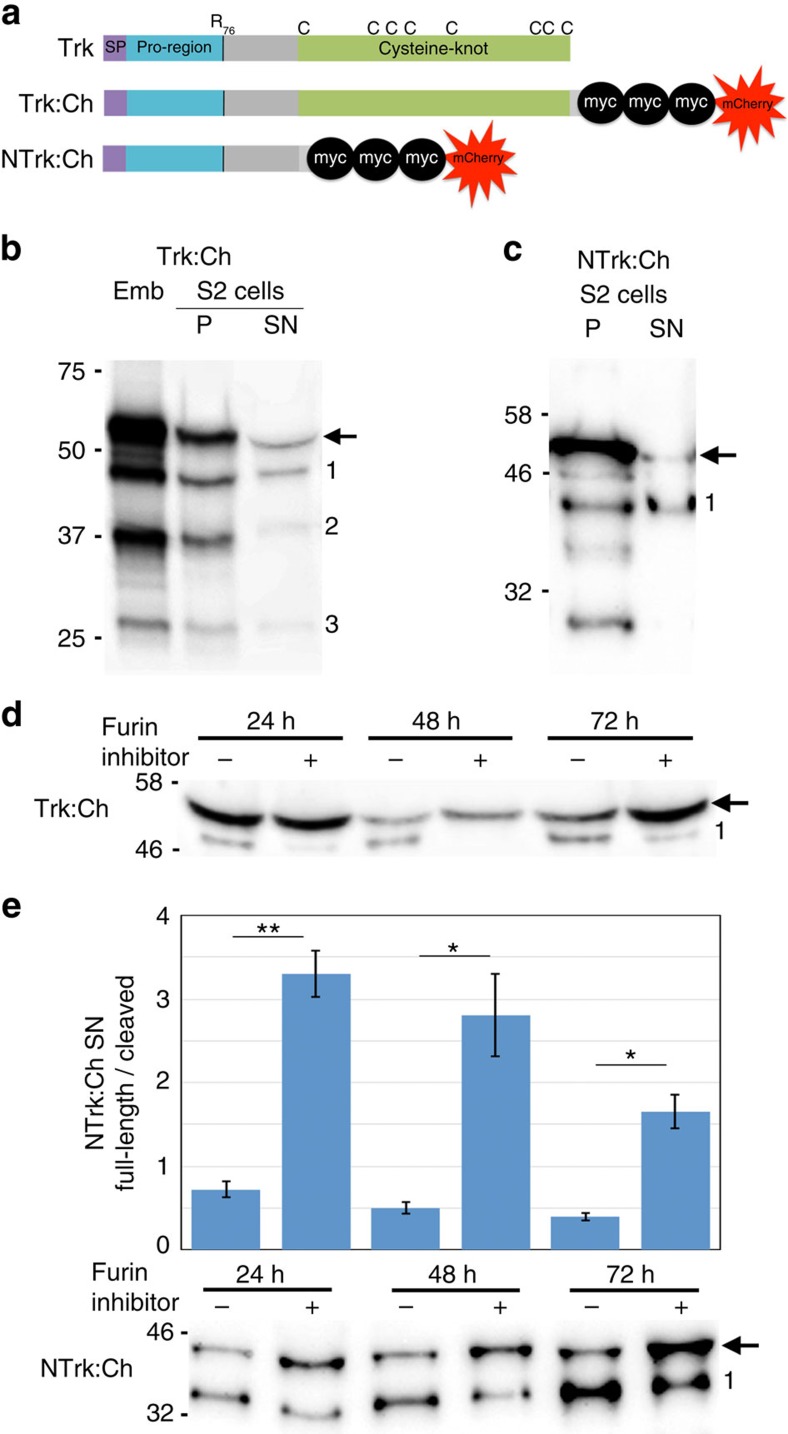
Furins cleave Trunk intracellularly. (**a**) Trk:Ch consists of the Trk coding sequence fused to three Myc epitopes and the fluorescent protein mCherry (Ch). NTrk:Ch is Trk:Ch with the cysteine-knot region removed. SP, signal peptide. (**b**) Immunoblot (anti-Myc) of Trk:Ch expression in embryos and S2 cells. Full-length Trk:Ch (predicted to be 55 kDa, arrowed), and three cleaved Trk:Ch species (1–3) are detectable in embryos (Emb) and both the S2 cell pellet (P) and supernatant (SN). (**c**) Immunoblot of NTrk:Ch expression in S2 cells. Full-length (predicted to be 44 kDa, arrowed) and cleaved (1) NTrk:Ch are detectable in the cell pellet and supernatant. (**d**) Immunoblot of supernatant from Trk:Ch-expressing S2 cells incubated with (+) and without (−) Furin inhibitors over 3 days. Trk:Ch cleavage is markedly reduced or absent when Furin inhibitor is present (compare species 1 level in lanes with and without inhibitor). For uncropped immunoblot see Supplementary Fig. 1a. (**e**) Immunoblot and quantification of NTrk:Ch in S2 cell supernatant. Cleavage is significantly reduced when Furin inhibitor is present (unpaired *t*-test, *P<0.05, **P<0.01). For uncropped immunoblot see Supplementary Fig. 1b. Error bars represent ±1 s.e. *n*=3 for each mean. Images are representative of at least three replicate immunoblots.

We first sought to determine the location of Trk cleavage. We noted that other cysteine-knot growth factors related to Trk, such as nerve growth factor-beta^15^ and bone morphogenetic protein 4 (ref. 16), are cleaved intracellularly. We reasoned that if Trk were to be similarly processed inside the cell then a role for Tsl in Trk cleavage would be unlikely as Tsl is known to be extracellular^7^,^8^,^9^,^12^. Since sampling the embryo and PVS separately for Trk cleavage products is technically challenging, we instead generated *Drosophila* S2 cells expressing a transgene encoding Trk tagged at the C-terminus with three Myc epitopes and the fluorescent protein mCherry (Trk:Ch, Fig. 1a). In both S2 cells and embryos expressing Trk:Ch, a protein of the expected size for full-length Trk:Ch and several Trk:Ch cleavage products were readily detectable (Fig. 1b), suggesting that the factors required for Trk processing in the embryo are present in S2 cells. Separation of the cells from the supernatant revealed the presence of Trk:Ch cleavage products in both fractions (Fig. 1b). Given that these cells express *tor* (Supplementary Fig. 1a), it was possible that the observed Trk:Ch cleavage products in the cell pellet could be the result of Trk:Ch binding to Tor on the cell surface. To address this, we deleted the Tor-binding region (C-terminal cysteine-knot) of Trk:Ch (NTrk:Ch, Fig. 1a) and performed the same experiment. NTrk:Ch cleavage products were also present in both the cell pellet and the supernatant (Fig. 1c). Together, these data suggest that Trk cleavage occurs intracellularly, before secretion.

### Furin proteases are required for Trk cleavage

Since our Trk:Ch-expressing cells recapitulated our observations of Trk cleavage *in vivo*, we were in a position to identify the protease(s) responsible for Trk cleavage. Previously, we have shown that the dibasic site at K75, R76 is critical for both Trk cleavage and function^14^. A search of the MEROPS database^17^ using KRSS (P2-P2′) as a substrate revealed the prohormone convertases, or Furins, as strong candidates for cleaving at this sequence. Furins are known to cleave and activate growth factors such as nerve growth factor-beta and bone morphogenetic protein 4 intracellularly during trafficking through the transgolgi network and secretory pathway (for review see ref. 18). To determine if Furins were responsible for Trk cleavage in S2 cells, we incubated the Trk:Ch- and NTrk:Ch-expressing cells with a mixture of Furin catalytic site inhibitor and Furin competitive substrate, as this has previously been shown to inhibit Furin activity in S2 cells^19^. This treatment resulted in a near-complete loss of cleavage product in the Trk:Ch cell supernatant (Fig. 1d and Supplementary Fig. 2a) and a significant reduction in the amount of cleavage product in the NTrk:Ch cell supernatant (Fig. 1e and Supplementary Fig. 2b). These data suggest that Furins cleave Trk in S2 cells.

### Furin 1 and Furin 2 act redundantly in terminal patterning

**Figure 2:**
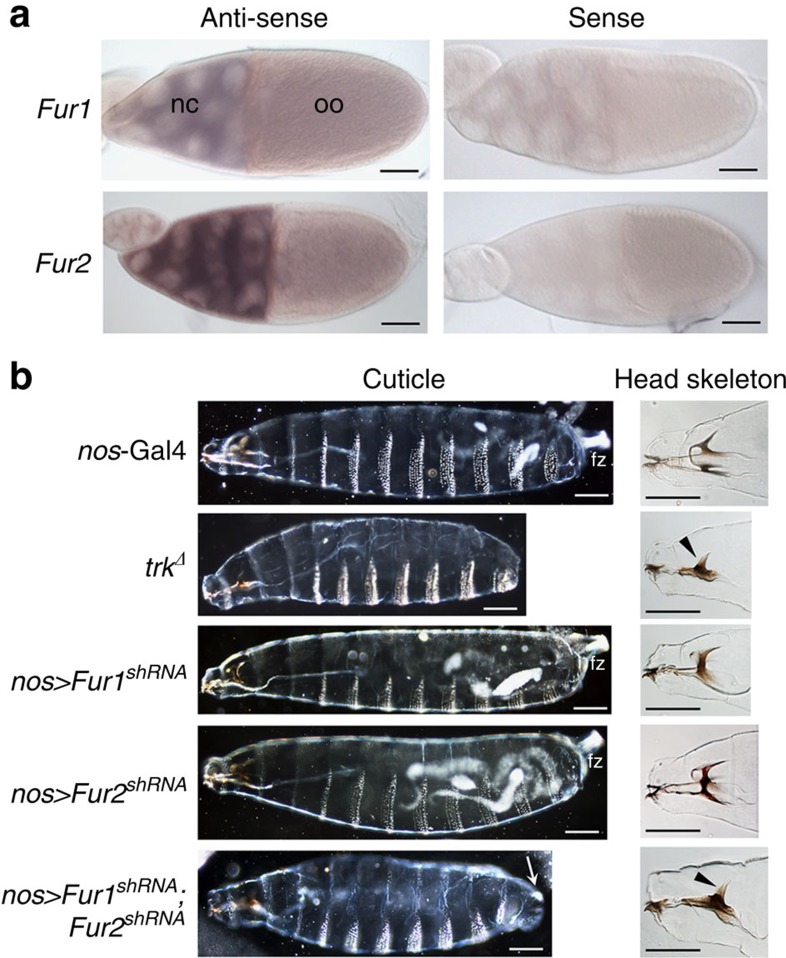
Furin1 and Furin2 are essential for terminal patterning. (**a**) RNA *in situ* hybridization for *Fur1* and *Fur2* in stage 10 egg chambers reveals both genes are expressed in the nurse cells (nc) and oocyte (oo). Sense controls show no staining. (**b**) Whole larval cuticles and head skeletons of animals with maternal knockdown (*nos*-Gal4) of *Fur1* and *Fur2* transcripts using UAS-shRNAs. Knockdown of either *Fur1* or *Fur2* alone has no effect. Co-knockdown of *Fur1* and *Fur2* results in terminal defects including a reduced posterior filzkorper (fz, arrowed) and head skeleton defects (arrowheads) similar to embryos laid by *trk*^Δ^ females. Anterior is to the left. Scale bars, 50 μm. Images are representative of at least two repeat experiments, with at least 100 individuals observed per genotype.

We next investigated whether Furin proteases are required for terminal patterning *in vivo*. The *Drosophila* genome contains three Furin-encoding genes: *Furin 1* (*Fur1*), *Furin 2* (*Fur2*) and *amontillado* (*amon*). Of these, we found only *Fur1* and *Fur2* were expressed in ovary tissue (Supplementary Fig. 1b). RNA *in situ* hybridization showed these genes to be expressed in the germline nurse cells (Fig. 2a). This suggests that, like *trk* and *tor*, *Fur1* and *Fur2* transcripts are deposited into the oocyte and present ubiquitously in the early embryo. To determine whether *Fur1* or *Fur2* were required for terminal patterning, we reduced their maternal expression by driving short-hairpin RNAs targeted against these genes with *nanos*-Gal4 (*nos*-Gal4). Knockdown of *Fur1* or *Fur2* alone produced mostly wild-type larvae (Fig. 2b). Strikingly however, knockdown of both genes resulted in a loss of terminal structures (Fig. 2b) as well as phenotypes consistent with dorso-ventral patterning defects (Supplementary Fig. 3). Taken together, these data demonstrate that Trk is cleaved intracellularly and that *Fur1* and *Fur2* act redundantly in Trk cleavage in terminal patterning.

### Extracellular Trk accumulation is dependent on Tsl

If Trk is activated ubiquitously by Furin cleavage before secretion from the embryo, how does Tsl, which is extracellular, restrict Tor signalling to the termini? It is possible that Tsl functions to bind and stabilize Trk to facilitate its accumulation in the PVS at the embryo poles. An alternative possibility is that, rather than being secreted ubiquitously into the PVS, Trk might instead be locally secreted at the embryo termini, and that Tsl plays a role in this process. In support of this idea, we note that pore-forming proteins have been previously shown to have the capacity to promote secretory events in eukaryotic cells^20^,^21^.

To test these ideas we aimed to determine the localization of Trk during processing and secretion into the PVS but before Tor binding. To do this we used the NTrk:Ch construct, as it lacks the Tor-binding region but is still processed and secreted in S2 cells. Expression of this transgene ubiquitously in the embryo with *nos*-Gal4 resulted in mostly wild-type embryos, however a small number of animals (3%) exhibited terminal defects (Supplementary Fig. 4a,b). Given that ubiquitous expression of wild-type Trk with *nos*-Gal4 has no phenotypic effect (Supplementary Fig. 4a), this suggests that NTrk:Ch may be competing with endogenous Trk for factors required for its function. Consistent with this idea, knockdown of either *Fur1* or *Fur2* strongly enhanced this dominant negative effect (Supplementary Fig. 4b), suggesting that NTrk:Ch may be sequestering Furin activity that is normally required for Trk cleavage. As the vast majority of embryos were wild type we were able to proceed with examining NTrk:Ch localization.

**Figure 3:**
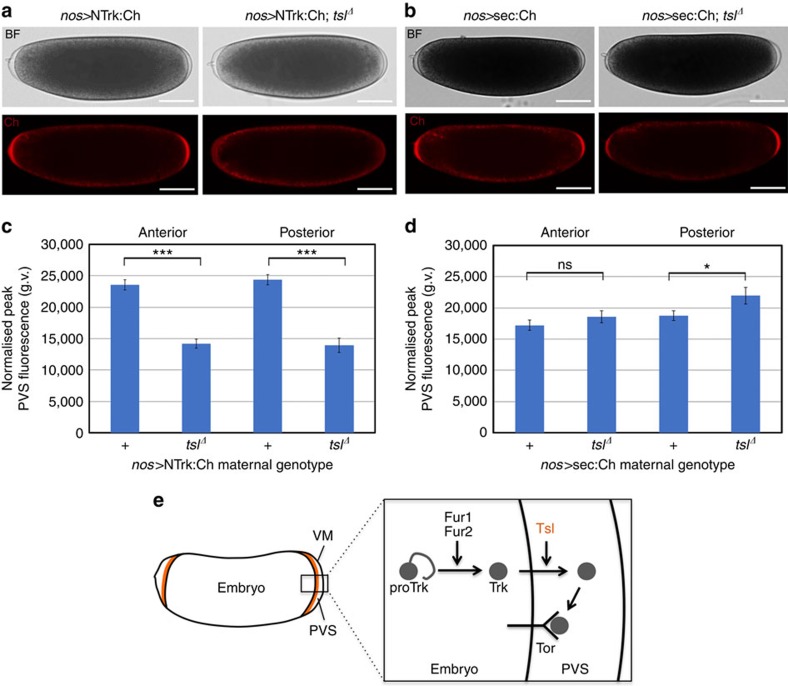
NTrk:Ch secretion is enhanced by Torso-like. (**a**) Strong accumulation of NTrk:Ch is observed in the polar PVS in <1 h wild type but not maternal *tsl* null mutant embryos (*tsl*^Δ^). Ch, mCherry. BF, brightfield. (**b**) sec:Ch accumulates in the polar PVS of wild-type and *tsl*^Δ^ embryos. Anterior is to the left. Scale bars, 100 μm. (**c**) The level of NTrk:Ch in the anterior and posterior PVS is markedly and consistently reduced in *tsl*^Δ^ embryos compared with wild type (unpaired *t*-test, ***P<0.0001, anterior: wild type *n*=197, *tsl*^Δ^ *n*=144; posterior: wild type *n*=185; *tsl*^Δ^ *n*=64). (**d**) Levels of sec:Ch are not reduced in the absence of Tsl compared with wild type (unpaired *t*-test, *P<0.05, anterior: wild type *n*=73, *tsl*^Δ^ *n*=57; posterior: wild type *n*=81; *tsl*^Δ^ *n*=54). ns, not significant. g.v., grey values. Error bars represent ±1 s.e. (**e**) Revised model for terminal patterning in *Drosophila*. Trk is activated by Furin cleavage within the embryo, possibly in the secretory pathway. Trk is then secreted at the embryo poles by a Tsl-dependent mechanism, and encounters the Tor receptor tyrosine kinase to initiate the signalling cascade required for terminal patterning.

Imaging of 1-h-old embryos confirmed that we could readily visualize NTrk:Ch in the PVS (Fig. 3a). Rather than being evenly distributed throughout the PVS, as might be expected if Trk was secreted uniformly from the embryo, we observed specific localization of NTrk:Ch in the PVS only at the embryo poles (Fig. 3a). To determine whether this localization pattern was caused by Trk secretion specifically at the termini, or simply the result of extracellular accumulation in the large polar PVS, we tested a transgene encoding a secretion signal peptide fused directly to mCherry (sec:Ch, ref. 22). sec:Ch also accumulated in the PVS at the poles (Fig. 3b), suggesting that the localization pattern of NTrk:Ch may be due to the increased PVS size at the poles.

Nonetheless, given that NTrk:Ch was clearly secreted into the PVS, we were able to investigate whether this extracellular accumulation was dependent on Tsl. Strikingly, in embryos expressing the NTrk:Ch protein in the absence of maternal Tsl, the amount of NTrk:Ch in the PVS was markedly reduced at both the anterior and posterior poles (unpaired *t*-test, *P*<0.001, Fig. 3a,c). Importantly, secretion of sec:Ch was not reduced in the absence of Tsl (Fig. 3b,d). Given that Tsl is localized specifically to the embryo poles, these data suggest that Tsl functions to enhance the extracellular accumulation of Trk in the PVS at the embryo termini.

## Discussion

Our data provide strong evidence that Trk is cleaved intracellularly by Fur1 and Fur2 before its secretion. The functional redundancy of these proteases in Trk processing explains why these enzymes were not identified in previous genetic screens. Since Trk cleavage occurs intracellularly, the extracellularly located Tsl is unlikely to be involved in controlling Trk cleavage events as previously proposed. In support of this idea, we discover that the role of Tsl is instead to enhance levels of extracellular Trk at the termini.

How might Tsl function to enhance extracellular Trk levels? One possible explanation is that Tsl binds to and stabilizes Trk at the embryo poles post-secretion, and this is necessary for generating the active Tor ligand. However, there are several lines of evidence against this idea. First, two independent groups have previously demonstrated that when Tor is artificially expressed only in the central region of the embryo, the active Tor ligand can readily diffuse from the poles and activate Tor in these regions, where Tsl is not present^3^,^6^. As several studies have shown that Tsl is physically associated with the embryo plasma membrane and the inner VM^7^,^8^,^9^,^12^, it seems unlikely that Tsl could diffuse and act away from the termini. Second, if the role of Tsl is to stabilize Trk by binding to it, then NTrk:Ch should be concentrated most where Tsl localizes, namely, on the inner VM and on the plasma membrane surface of the embryo^7^,^8^,^9^,^12^. In contrast, we observe NTrk:Ch homogenously distributed throughout the polar PVS, and a general reduction throughout the PVS in *tsl* null mutants. Finally, if Tsl was required for Trk stability we might expect to see decreased levels of full-length Trk protein and increased degradation in *tsl* null mutants. However, in a previous study we did not observe any difference in levels of full-length Trk or its cleavage pattern in *tsl* mutants^14^.

We thus favour an alternative model whereby Tsl facilitates the secretion of Trk (Fig. 3e). This idea is based on the fact that Tsl is a member of the membrane attack complex/perforin-like/bacterial cholesterol-dependent cytolysin (MACPF/CDC) protein superfamily^13^. These proteins are best characterized for their pore-forming and membrane-damaging activities in immunity and defence across many taxa^23^,^24^. However, some MACPF/CDC proteins can trigger defence-related secretory events in eukaryotic cells^20^,^21^. As several studies have shown that Tsl is associated with the embryonic plasma membrane^8^,^9^, it is possible that it promotes Trk secretion via a pore-forming or membrane-damaging mechanism. Further studies will be necessary to determine if this is the case.

Finally, we note that Tsl has an additional key role in the control of larval growth and developmental timing^25^,^26^. Given that this activity is independent of Trk, we reason that Tsl may influence the secretion of other growth factors in the fly. Furthermore, several mammalian perforin-like proteins play critical but poorly understood roles during development^27^,^28^. The control of growth factor secretion may therefore be a general role of perforin-like proteins in eukaryotes.

## Methods

### Drosophila stocks

The following stocks were used: *w*^*1118*^(BL5905), *Gal4::VP16-nos.UTR* (BL7293), *ZH-86Fb* (BL24749, ref. 29) and germline shRNAs for *Fur1* (BL42481) and *Fur2* (BL51743), from the Bloomington *Drosophila* stock centre, UASp-sec:Ch (gift from Stefano De Renzis^22^), *tsl*^Δ^ (ref. 25) and *trk*^Δ^ (ref. 14), null mutants of *tsl* and *trk*, respectively. All flies were maintained on standard media at 25 °C.

### Trk constructs and generation of transgenic lines

The Trk:Ch construct encodes the entire Trk coding sequence followed by a short linker peptide (SAGSAS), three tandem Myc epitopes, and the mCherry fluorescent protein (Fig. 1a). The NTrk:Ch is different to Trk:Ch in that it lacks residues 143–235 of the Trk coding sequence. Trk:Ch and NTrk:Ch were synthesized (Genscript) and cloned into the NotI/XbaI sites of the pAc5.1/V5-his vector (Life Technologies). To generate flies expressing NTrk:Ch, this was also ligated into the NotI/XbaI sites in pUASp (ref. 30). For transgenesis, *w*^*1118*^ embryos were injected (BestGene) using standard P-transformation methods and several independent transgenic lines were established and tested.

### S2 cell Furin inhibitor assay

*Drosophila* S2 cells (SG4 line, from the *Drosophila* Genomics Resource Centre) were cotransfected with 0.4 μg of Trk:Ch or NTrk:Ch plasmids together with pCoHygro (for generation of stable lines) using Effectene transfection reagent (Qiagen). To test for Trk cleavage in the cells, only fresh cells (unfrozen) were used. The Furin inhibitor assay was performed as previously described^19^. Cells were collected by brief centrifugation, washed once with phosphate buffered saline, and resuspended in sufficient fresh media (Schneiders insect cell media and 10% fetal bovine serum) for ∼75% confluence. Cells were aliquoted into wells of a 96-well plate (Greiner bio-one), and incubated with either a mixture of two Furin inhibitors, a Furin active site blocker (Calbiochem 344930) and a polyarginine compound that acts as a potent competitive inhibitor of Furin activity (Calbiochem 344931), at a final concentration of 50 μM each, or with a solution containing only dimethylsulphoxide diluted accordingly (mock inhibitor). Supernatants were separated from the cells and snap frozen at *t*=24, 48 and 72 h, respectively. Each experiment was performed with three biological replicates.

### Immunoblotting

Fresh cell pellets were washed twice in milliQ H_2_O before being ground in 100 μl lysis buffer (50 mM Tris-HCl (pH 7.5), 150 mM NaCl, 2.5 mM EDTA, 0.2% Triton-X, 5% glycerol, complete EDTA free protease inhibitor cocktail (Roche)). For immunoblotting of embryos, ∼50 μl volume of 0–4-h-old embryos were homogenized in 200 μl lysis buffer and spun at 500*g* for 5 min at 4 °C. Reducing buffer containing 6 M urea was added to all samples before boiling and separation by SDS–PAGE (any kDa TGX, Biorad) followed by transfer onto an Immobilon-P membrane (Millipore). Membranes were probed with anti-Myc (1:1,000, Cell Signalling; 9B11), washed and incubated with HRP-conjugated secondary antibody (1:10,000, Southern Biotech). Immunoblots were developed using ECL prime (GE healthcare) and imaged using a chemiluminescence detector (Vilber Lourmat). Blot images were quantified where necessary using ImageJ and treatment differences determined by unpaired *t*-tests from at least three biological replicates.

### Cuticle preparations

Adults were allowed to lay on a media containing apple juice supplemented with yeast paste for 24 h before being removed. Embryos were left to develop for a further 24 h before dechorionation and mounting on slides in a mixture of 1:1 (vol/vol) Hoyer's solution: lactic acid. Slides were incubated overnight at 65 °C and imaged using dark field optics (Leica).

### Imaging and PVS fluorescence quantification

For imaging and image quantification, 0–1.5-h-old embryos were collected and dechorionated in 50% bleach (vol/vol) for 1.5 min before being mounted flat in 8-well slides (Ibidi) and covered in PBS. Two wells (upper and lower) were used for each imaging session with each genotype allocated to its well at random. Embryo image data were collected on both brightfield (LED transmission) and red (laser excitation 561 nm) channels through their entire *z* axis using a spinning disk confocal microscope (Olympus CV1000) and a × 10 dry objective. In a single session, ∼20 embryos of each genotype were captured. Microscope settings remained unchanged for the entire experiment and fluorescence maxima were never above the detection limit of the camera. Following image collection, genotypes were noted and all quantification were performed blind. Embryos without an obvious PVS and those beyond stage two (∼1-h-old, after polar bud formation) were excluded from further analyses. For each of the remaining embryos, a single *z* plane where the PVS was in sharpest focus was extracted, rotated and cropped. For quantification, a window 15 pixels high and spanning the length of the embryo (anterior–posterior) was drawn over the centre of each embryo. The average fluorescence (grey value) for each pixel along the embryo length within this window was then extracted and the peak PVS fluorescence determined separately for anterior and posterior poles (Supplementary Fig. 5). To normalize against background intensity variation between embryos, the average fluorescence across a 100-pixel window placed within the middle of each embryo was subtracted from the peak heights. For each genotype mean, between 54 and 197 PVS compartments were quantified. Unpaired *t*-tests were used to determine whether genotypes were significantly different.

### RNA *in situ* hybridisations

RNA *in situ* hybridization on whole-mount dissected and fixed (4% paraformaldehyde) ovaries was performed using DIG-labelled sense and anti-sense RNA probes transcribed from pGEMT-Easy (Promega) clones of *Fur1* (750 bp, F-5′-CGACGTCGTGCGATGGAGCAGGCA-3′, R-5′-CGAATCCGTGATCCTTGGCAACCG-3′) and *Fur2* (650 bp, F-5′-ATACGACGAGCTCCAGCCGAGTTA-3′, R-5′-GAAGGCGCCAAACATATCGACCCC-3′) following standard protocols^31^. Briefly, probes were hybridized to tissue overnight at 55 °C and washed in hybridization buffer (4 × saline sodium citrate buffer, 50% vol/vol formamide, 0.1% vol/vol Tween-20, 50 μg ml^−1^ heparin) for 36 h before incubation with alkaline phosphatase conjugated anti-digoxygenin and colour development with 5-bromo-4-chloro-3-indolyl phosphate and nitro blue tetrazolium chloride. Imaging was performed under differential interference contrast optics on a Leica DM LB compound microscope.

### Ovary and S2 cell reverse transcription-PCR

Ten pairs of ovaries or ∼20 μl of pelleted S2 cells were snap frozen before RNA was extracted using TRIsure reagent (Bioline) and treated with DNAse (Promega). Complementary DNA was generated using Tetro reverse transcriptase (Bioline) by priming 5 μg RNA with oligo-dT and random hexamers. Reverse transcription-PCR was performed using Go-taq flexi DNA polymerase (Promega) using primers specific for *Fur1* (F-5′-TCAATCCGTAGCCAAAGGAG-3′, R-5′-TTCGCACATCACAAGGTCTC-3′), *Fur2* (F-5′-TTCCGGATCCGTTGTTTAAG-3′, R-5′-CCACCAAGTATTGCATGTCG-3′), *amon* (F-5′-GAACCCGAATAGGTGGTTGA-3′, R-5′-CCACATATACAGCGCCTCCT-3′), *tor* (F-5′-TGCTTGGATTGGTATCCCTAT-3′, R-5′-TGGGTCACAGTAAGATTCTCT-3′) and a control gene, *Rp49* (F-5′-CAGTCGGATCGATATGCTAAGCT-3′, R-5′-TAACCGATGTTGGGCATCAGATA-3′).

## Additional information

**How to cite this article:** Johnson, T. K. *et al.* Torso-like mediates extracellular accumulation of Furin-cleaved Trunk to pattern the Drosophila embryo termini. *Nat. Commun.* 6:8759 doi: 10.1038/ncomms9759 (2015).

## Supplementary Material

Supplementary Information Supplementary Figures 1-5 and Supplementary References
