# Supplementary material for: Torso-like mediates extracellular accumulation of Furin-cleaved Trunk to pattern the Drosophila embryo termini
Source: Nat Commun. 2015 Oct 28;6:8759. doi: 10.1038/ncomms9759 (PMC4640135; doi:10.1038/ncomms9759)
Supplement: Supplementary Information — Supplementary Figures 1-5 and Supplementary References [file ncomms9759-s1.pdf]

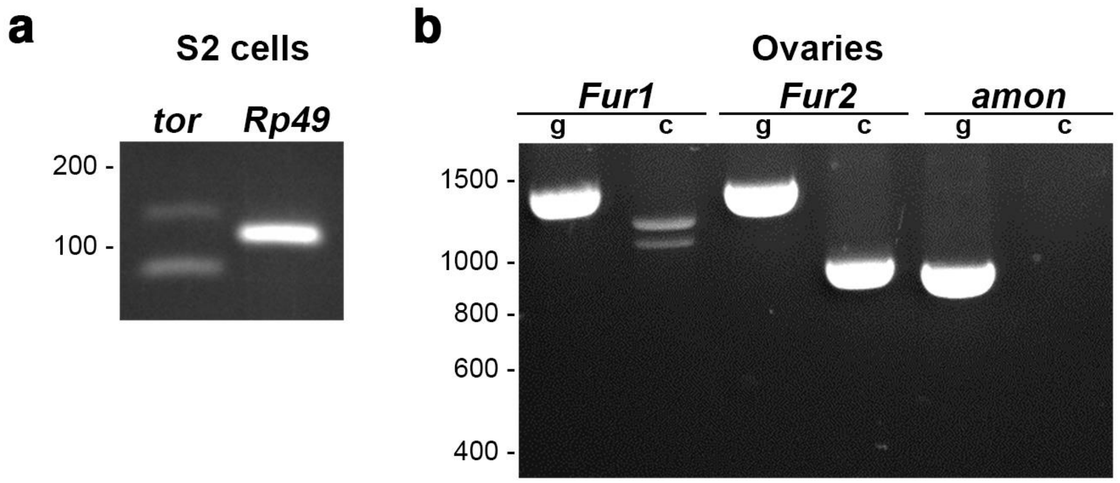

Supplementary Figure 1. Expression of *torso* and *Furin* genes in S2 cells and ovaries respectively. (**a**) RT-PCR of *tor* from S2 cell cDNA. The two bands represent the *tor* transcript (71bp) and genomic sequence (139bp). Rp49 is a control gene (110bp, cDNA only). (**b**)*Fur1* and *Fur2* but not *amon* are expressed in ovaries. Primers are intron spanning g, genomic DNA. c, cDNA. For *Fur1:* expect 1371bp (g), 1138bp (c). *Fur2:* expect 1359bp (g), 889bp (c). *amon:* expect 866bp (g), 550bp (c).

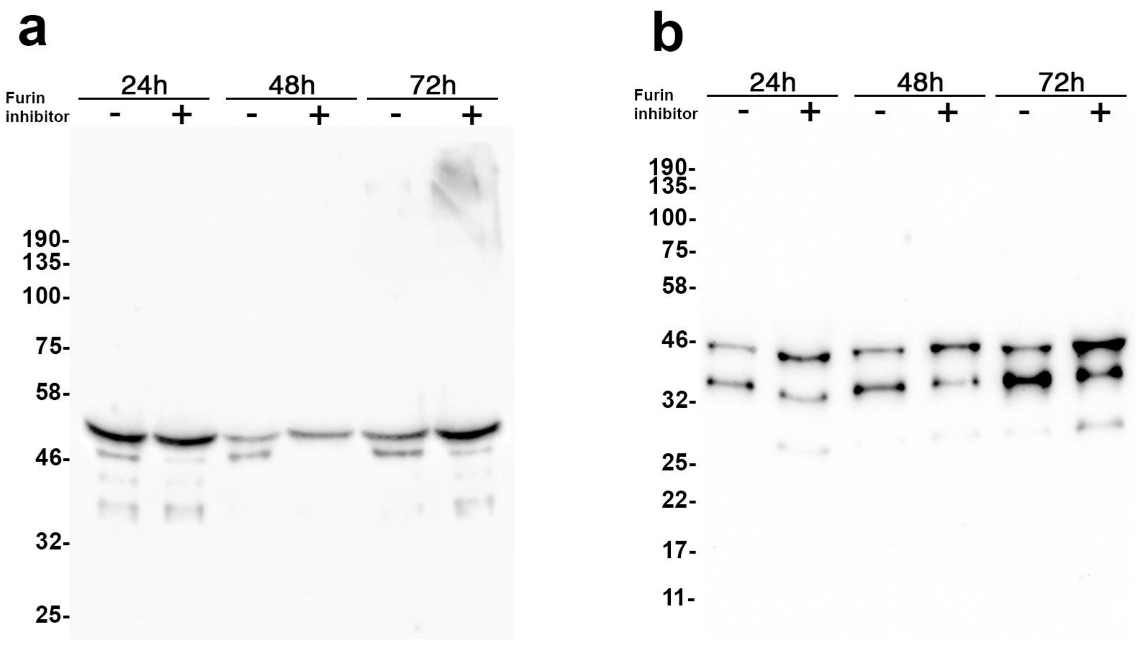

Supplementary Figure 2. Uncropped immunoblots from Furin inhibitor experiments.

(**a**) Uncropped immunoblot from Fig. 1d. (**b**) Uncropped immunoblot from Fig. 1e.

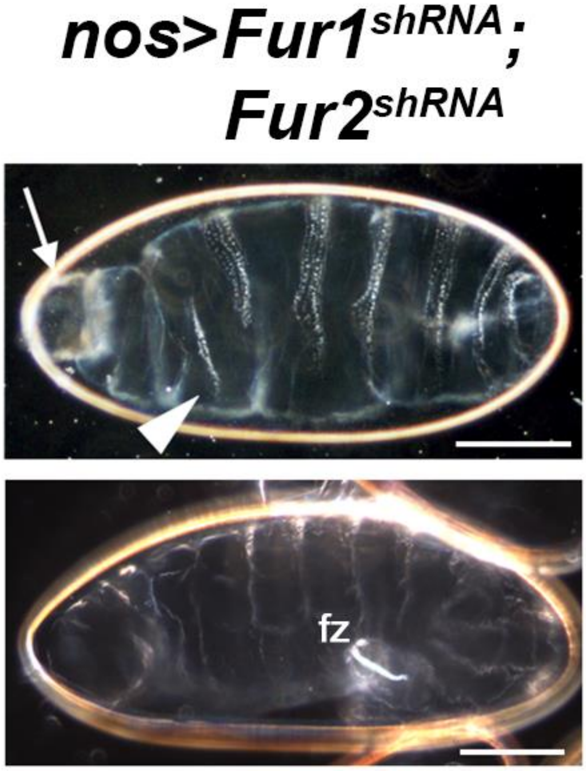

Supplementary Figure 3. Co-knockdown of *Fur1* and *Fur2* results in dorso-ventral patterning defects. In addition to terminal patterning defects, lateral denticle belt fields (arrowhead) indicating mild ventralisation and externalised head skeletons (arrowed) that indicate head involution defects are commonly observed (top panel). Germband retraction failure evident by the centrally located filzkorper (fz) is also observed (bottom panel). These phenotypes resemble reduced activity of the *Drosophila* BMP4 homolog Decapentaplegic[1]. Decapentaplegic is required for embryonic dorso-ventral patterning and is known to be cleaved by Fur1 and Fur2 during wing development and in S2 cells[2]. Scale bars, 100 μm.

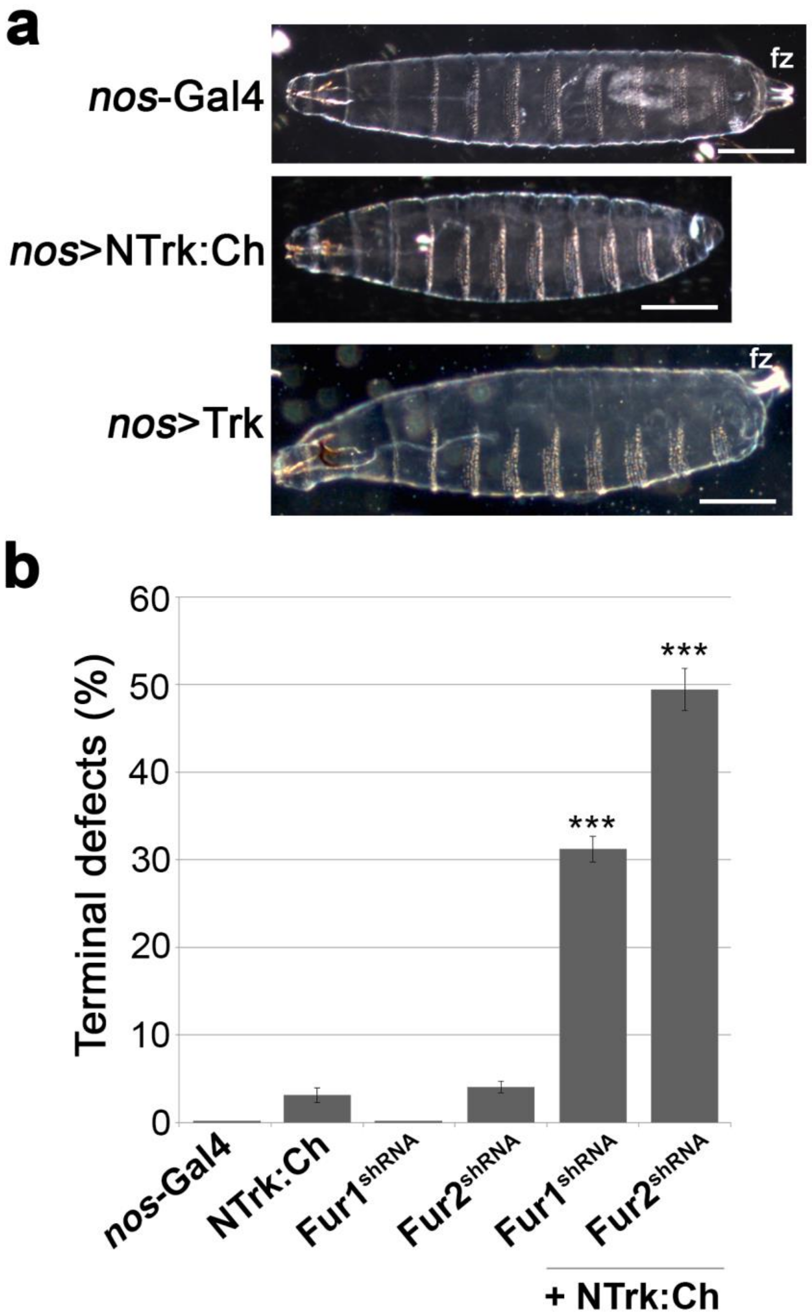

Supplementary Figure 4. NTrk:Ch competes with endogenous Trunk for Furin activity. (**a**) Expression of NTrk:Ch with *nanos*-Gal4 results in weak terminal patterning defects (fz, filzkorper) while expression of wildtype Trk has no effect. Scale bars, 100 µm. (**b**) Maternal knockdown of either *Fur1* or *Fur2* with *nanos*-Gal4 significantly enhances the NTrk:Ch dominant negative effect (unpaired t-test, ***$p<0.001$). Error bars represent ±1 standard error. n=3 for each mean, with at least 100 embryos scored in per replicate.

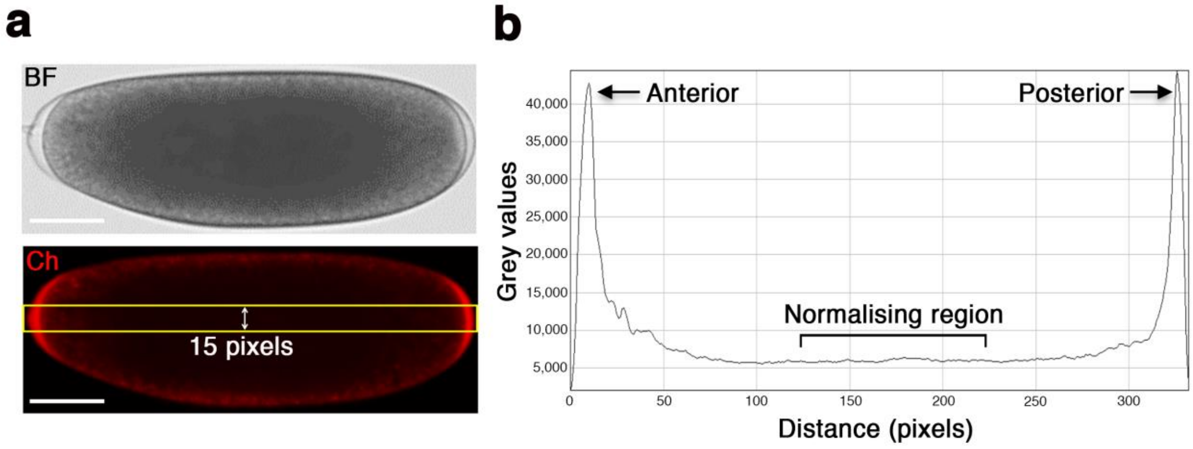

Supplementary Figure 5. Quantification of NTrk:Ch in the polar perivitelline space. (**a**) For each embryo, images were captured on both brightfield (BF) and Ch channels. Following orientation (anterior-posterior) and cropping, a 15-pixel high window was drawn across the length of the embryo and fluorescence values within extracted. Scale bars, 100 µm. (**b**) Plot of the grey values (fluorescence intensities) within the window along the length of the embryo. Anterior and posterior peak PVS values were taken following subtraction of the average value for the normalising region to account for variation between embryos.

Supplementary References

1 Irish, V.F. & Gelbart, W.M. The *decapentaplegic* gene is required for dorsal-ventral patterning of the *Drosophila* embryo. *Genes Dev.* **1**, 868-879 (1987)

2 Künnapuu, J., Björkgren, I. & Shimmi, O. The *Drosophila* DPP signal is produced by cleavage of its proprotein at evolutionary diversified furin-recognition sites. *Proc. Natl. Acad. Sci. USA* **106**, 8501-8506 (2009)
